# Biosafety of a novel covered self-expandable metal stent coated with poly(2-methoxyethyl acrylate) *in vivo*

**DOI:** 10.1371/journal.pone.0257828

**Published:** 2021-09-24

**Authors:** Tetsuya Ishizawa, Naohiko Makino, Yasuharu Kakizaki, Akiko Matsuda, Yoshihide Toyokawa, Shun Ooyama, Masaru Tanaka, Yoshiyuki Ueno

**Affiliations:** 1 Faculty of Medicine, Department of Gastroenterology, Yamagata University, Yamagata, Japan; 2 Piolax Medical Devices, Inc., Kanagawa, Japan; 3 Frontier Center for Organic Materials, Yamagata University, Yamagata, Japan; 4 Soft Materials Chemistry, Institute for Materials Chemistry and Engineering, Kyushu University, Fukuoka, Japan; Shanghai Jiao Tong University Medical School Affiliated Ruijin Hospital, CHINA

## Abstract

Covered self-expandable metal stents (CSEMS) are often used for palliative endoscopic biliary drainage; however, the unobstructed period is limited because of sludge occlusion. The present study aimed to evaluate the biosafety of a novel poly(2-methoxyethyl acrylate)-coated CSEMS (PMEA-CSEMS) for sludge resistance and examine its biosafety *in vivo*. Using endoscopic retrograde cholangiopancreatography, we placed the PMEA-CSEMS into six normal porcine bile ducts and conventional CSEMS into three normal porcine bile ducts. We performed serological examination and undecalcified histological analysis at 1, 3, and 6 months during follow-up. In the bile ducts with PMEA-CSEMS or conventional CSEMS, we observed no increase in liver enzyme or inflammatory marker levels in the serological investigations and mild fibrosis but no inflammatory response in the histopathological analyses. Thus, we demonstrated the biosafety of PMEA-CSEMS *in vivo*.

## Introduction

Curative resection can be performed in <20% of patients with pancreatobiliary cancer [1]. Palliative endoscopic biliary drainage has been widely utilized for patients with malignant biliary obstruction to improve their quality of life [2]. Self-expandable metal stents have a longer patency than plastic stents [3]; however, the long-term patency of the former is limited.

Covered self-expandable metal stents (CSEMS) were developed to prevent stent occlusion due to tumor ingrowth and epithelial hyperplasia [1]; however, they often become occluded with sludge [4]. This occlusion mechanism has been described as follows: after insertion, (1) host proteins rapidly cover the stent’s inner surface, (2) bacteria adhere to the proteins, (3) these bacteria form viscous biofilms, and (4) the biofilms engulf foreign bodies, such as dietary fibers, causing an increase in thickness [5]. Therefore, we believe that sludge formation could be reduced by inhibiting the adsorption of proteins attached to the stent surface.

The poly(2-methoxyethyl acrylate) (PMEA) polymer reportedly inhibits protein and platelet adsorption [6]. Presumably, PMEA forms a layer of water on the surface [7]. We hypothesized that PMEA-coated CSEMS will reduce sludge formation by inhibiting protein adsorption. An in vitro study revealed that PMEA-coated CSEMS suppressed early stage biliary sludge formation [8]. In this study, we aimed to evaluate the biosafety of PMEA-coated CSEMS for sludge resistance in a porcine model.

## Materials and methods

### Stents

A PMEA-CSEMS and an uncoated CSEMS (conventional CSEMS) were prepared as previously reported [8]. Each CSEMS was 60-mm long and had a 6-mm inner diameter. Uncovered self-expandable metal stents (USEMS; length, 40 and 60 mm and inner diameter, both 6 mm) were prepared to prevent the migration of the CSEMS or the control.

### Animal care and stenting

**Fig 1:**
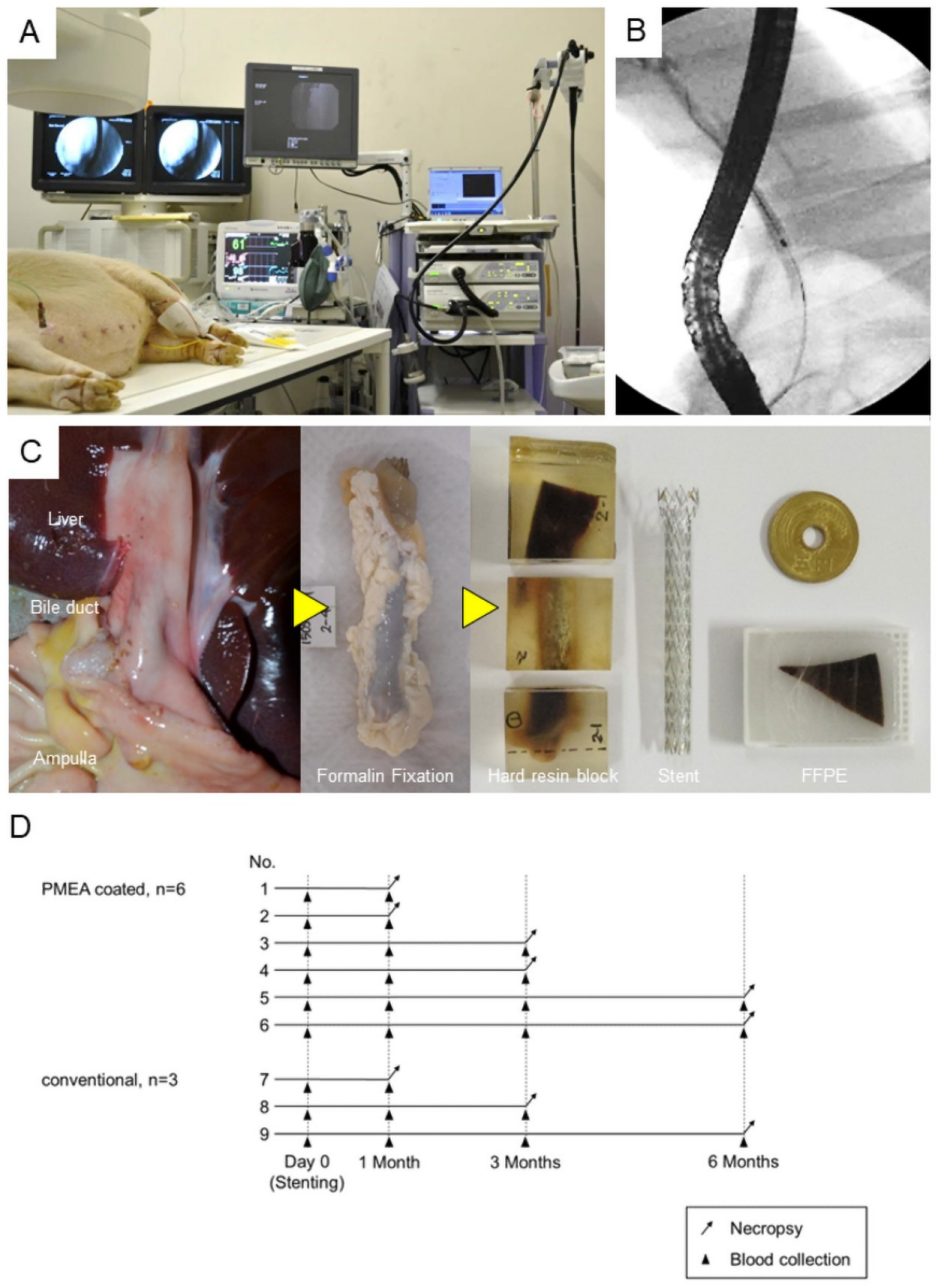
Methods. A. Treatment room layout. B. Endoscopic stenting in the normal bile duct. C: Flow from necropsy to pathological specimen preparation. d: Flow from stent placement to necropsy.

Eleven female Göttingen mini-pigs (*Sus scrofa domesticus*, Ellegaard, Denmark) were maintained, including their acclimatization periods, at the Laboratory Animal Center, Institute for Promotion of Medical Science Research, Yamagata University Faculty of Medicine, Japan. The age of the pigs (mean ± standard deviation) was 16.6 ± 3.9 months and body weight (mean ± standard deviation) was 35.4 ± 4.1 kg. Each pig was housed in a singular cage at a room temperature of 22°C and fed standard chow and water. All the pigs were healthy and specific-pathogen-free and had no genetic modification and previous procedures. Endoscopic procedures were performed between 10:00 am and 4:00 pm in the radiography room of the Laboratory Animal Center. The pigs were fasted on the morning of the endoscopic procedure and anesthetized using intramuscular medetomidine (0.05 mg/kg), midazolam (0.5 mg/kg), and butorphanol (0.5 mg/kg) for a smooth introduction and stable depth of anesthesia [9]; anesthesia was maintained using isoflurane. Blood samples were collected before the endoscopic procedure. Vital signs were monitored during the procedure (Fig 1A). The stents were placed in the normal bile duct using endoscopic retrograde cholangiopancreatography (ERCP) (Fig 1B). This was concerning owing to the possibility of migration of the metal stent because the normal bile duct showed no stenosis. In the pilot study, the CSEMS migrated from the bile duct (data not shown). To prevent migration of the CSEMS, a short (40-mm long) USEMS with flare was first placed in the normal bile duct, followed by placement of a CSEMS (PMEA-CSEMS or conventional CSEMS) in the USEMS. Alternatively, a 60-mm long USEMS was placed in a 40-mm long USEMS with flare to investigate the effect of USEMS. In addition, a control without ERCP was prepared. After performing the procedures, atipamezole hydrochloride (0.2 mg/kg) was intramuscularly injected as an anesthetic antagonist, and the pigs were carefully monitored until recovery. After the endoscopic procedure, the dietary intake and general condition of the pigs were monitored. No criteria were set for including and excluding animals during the experiment. No randomization was used to allocate the experimental units to the control and treatment groups, and confounders were not controlled. The sample size was determined with reference to the study that evaluated safety and efficacy of a plastic stent coated with stone-dissolving agents in a porcine model (5 treatments vs. 5 controls) [10]. No blinding was applied in this study.

### Sample collection

Blood collection and necropsy were performed at 1, 3, and 6 months after stent placement (Fig 1C and 1D). The liver and bile duct were removed and fixed in a formalin solution (Fig 1C). The pathological specimens of the bile duct were then prepared using undecalcified hard resin blocks (Sept Sapie Co., Ltd., Japan; Fig 1C). Cole’s hematoxylin–eosin staining [11] was performed on two undecalcified sections on the liver side and the ampulla side of the bile duct (Sept Sapie Co., Ltd.). The pathological specimens of the liver were embedded in paraffin (Fig 1C) and stained with hematoxylin and eosin.

### Blood biochemical and pathological evaluations

Blood biochemistry comprised measurement of glutamic pyruvic transaminase (GPT), alkaline phosphatase (ALP), and albumin (ALB) levels as well as white blood cell (WBC) count. GPT (L-alanine substrate/leuco dye method), ALP (p-nitrophenyl phosphate substrate method), and ALB (bromocresol green method) were measured using DRI-CHEM NX500V (Fujifilm Corp., Tokyo, Japan). The WBC count was measured using VetScan HM2 (ABAXIS, California, USA). Pathological findings in the bile duct and liver were evaluated according to the four stages of inflammatory cell infiltration and fibrosis: none, mild, moderate, and severe. Infiltration of small round cells, such as lymphocytes and plasma cells, bleeding, granulation tissue proliferation, presence or absence of foreign body giant cells, and proliferation of bile duct mucosal epithelial cells and submucosal muscles were evaluated. No criteria were set for including and excluding animals during the data analysis. The primary outcome was the degree of inflammatory cell infiltration to the bile duct wall.

### Statistical analysis

Statistical analysis was performed using EZR version 1.40 (Easy R) [12]. The Mann–Whitney *U* test was used for comparisons between the two groups. The Kruskal–Wallis test was used for comparisons of three or more discontinuous variables. Differences between the groups were considered statistically significant at p <0.05. We did not evaluate whether the data met the assumptions of the statistical approach because of a biosafety test.

### Ethical considerations

All treatment protocols were approved by the Yamagata Animal Experiment Committee (approval numbers: 26161 and 27117 in 2015, 28034 in 2016, and 29020 in 2017) and performed according to the Regulation of Animal Experiments of Yamagata University.

## Results

The PMEA-CSEMS were placed in six of the nine pigs, the conventional CSEMS were placed in three pigs, the USEMS was placed in one pig, and no stent was placed in another pig. The six animals with PMEA-CSEMS were divided into three groups of two pigs each, and a different group underwent blood collection and necropsy under the same sedation conditions as those for pretreatment at 1, 3, and 6 months (PMEA-CSEMS group). Each of the three pigs with conventional CSEMS underwent the same procedures as those performed in the PMEA-CSEMS group at 1, 3, and 6 months (conventional CSEMS group; Fig 1D). No adverse events were observed in the PMEA-CSEMS and conventional CSEMS groups during the follow-up period. However, the pig that received the USEMS died of obstructive jaundice 20 days after ERCP.

**Fig 2:**
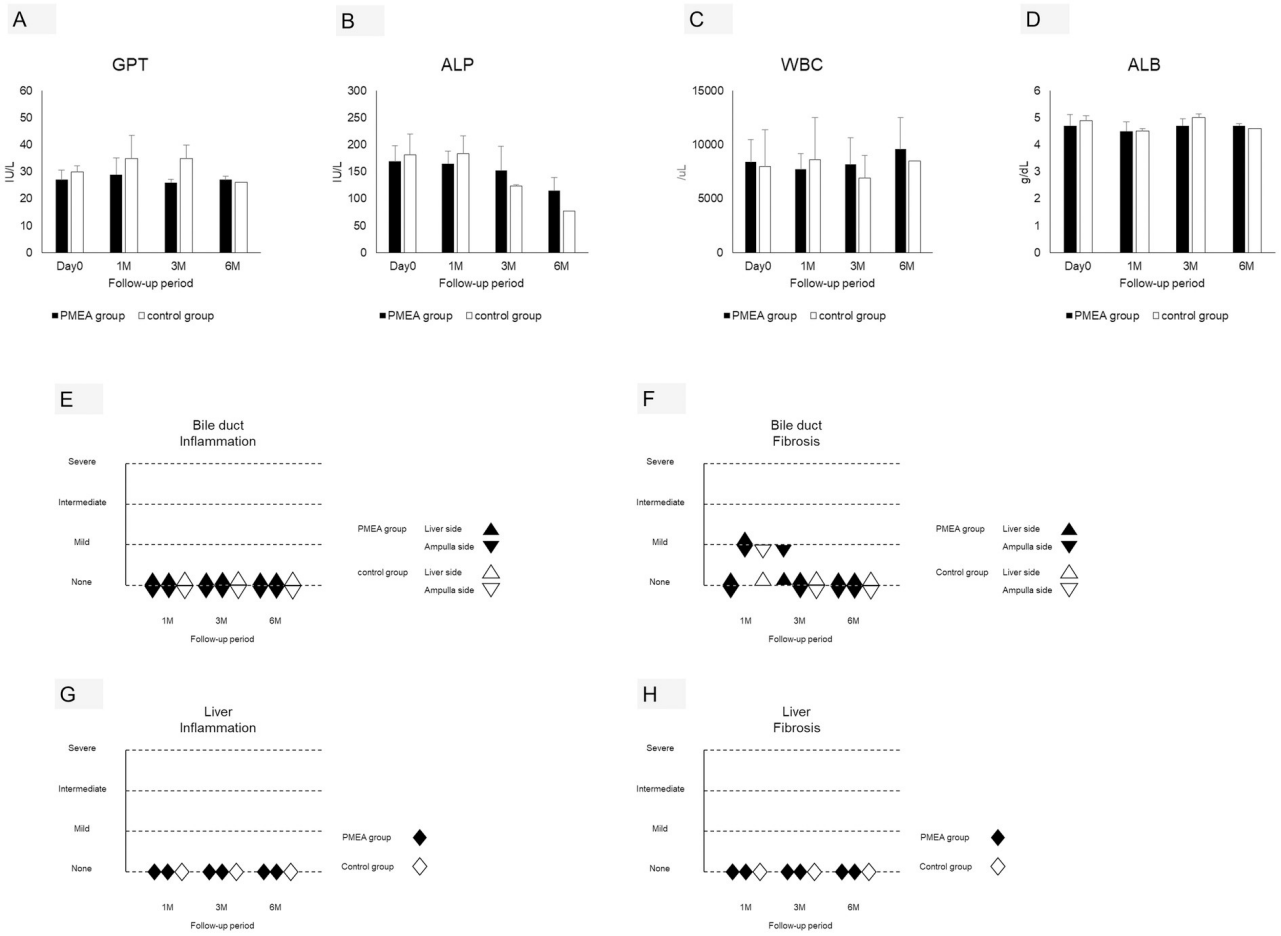
Blood biochemical and pathological evaluations of the PMEA and conventional CSEMS groups. A–D: No increase in GPT, ALP, and ALB levels or in the WBC count was observed in either of the groups at any of the follow-up time points. E–H: Nearly no pathological changes were observed in either of the groups at follow-up. GPT, glutamic pyruvic transaminase; ALP, alkaline phosphatase; WBC, white blood cell; ALB, albumin.

In the blood biochemistry evaluation, no significant increases in GPT, ALP, and ALB levels or WBC count were observed at any of the follow-up time points in the PMEA-CSEMS (n = 6) and conventional CSEMS groups (n = 3; Fig 2A–2D, Tables 1, 2 and S1 Table). Compared with the PMEA-CSEMS and conventional CSEMS groups, the USEMS group showed significant increases in GPT and ALP levels at 20 days (GPT, 119 IU/dL; ALP, 927 IU/dL; S1 Fig and S2 Table). Compared with the PMEA-CSEMS and conventional CSEMS groups, the control group without ERCP showed no significant increases in GPT, ALP, and ALB levels and WBC count (GPT, 22 IU/L; ALP, 106 IU/L; WBC, 7090/μL; 4.8 g/dL; S1 Fig and S2 Table).

**Table 1:** Statistical analysis of the blood biochemical evaluation results of the PMEA-CSEMS and conventional CSEMS groups.

Group	Unit	D0	1M	3M	6M	P value	Statistical method
PMEA-CSEMS	GPT (IU/L), Ave (SD)	26.5 (3.5)	29 (6.2)	26.3 (1.0)	27 (1.4)	0.898	
	ALP (IU/L), Ave (SD)	169 (29)	165 (23)	152 (37)	115 (25)	0.194	
	WBC (/dL), Ave (SD)	8300 (2100)	7730 (1500)	8170 (2500)	9600 (2900)	0.779	
	ALB (g/dL), Ave (SD)	4.7 (0.4)	4.5 (0.4)	4.7 (0.2)	4.7 (0.1)	0.733	Kruskal–Wallis test
Conventional CSEMS	GPT (IU/L), Ave (SD)	29.7 (2.1)	34.7 (8.4)	34.5 (5.0)	26 (0)	0.563	
	ALP (IU/L), Ave (SD)	181 (39)	184 (32)	124 (2.1)	77 (0)	0.132	
	WBC (/dL), Ave (SD)	8010 (3400)	8590 (3900)	6930 (2100)	8520 (0)	0.844	
	ALB (g/dL), Ave (SD)	4.9 (0.2)	4.5 (0.1)	5.0 (0.1)	4.6 (0)	0.0939	Kruskal–Wallis test

**Table 2:** Statistical analysis of the blood biochemical evaluation results for each follow-up period of the PMEA-CSEMS and conventional CSEMS groups.

Unit	Period	PMEA-CSEMS	Conventional CSEMS	P value	Statistical method
GPT (IU/L)	D0, Ave (SD)	26.5 (3.5)	29.7 (2.1)	0.185	Mann–Whitney *U* test
	1M, Ave (SD)	29 (6.2)	34.7 (8.4)	0.697	
	3M, Ave (SD)	26.3 (1.0)	34.5 (5.0)	0.1	
	6M, Ave (SD)	27 (1.4)	26 (0)	1	
ALP (IU/L)	D0, Ave (SD)	169 (29)	181 (39)	0.694	Mann–Whitney *U* test
	1M, Ave (SD)	165 (23)	184 (32)	0.262	
	3M, Ave (SD)	152 (37)	124 (2.1)	0.348	
	6M, Ave (SD)	115 (25)	77 (0)	0.667	
WBC (/dL)	D0, Ave (SD)	8300 (2100)	8010 (3400)	1	Mann–Whitney *U* test
	1M, Ave (SD)	7730 (1500)	8590 (3900)	1	
	3M, Ave (SD)	8170 (2500)	6930 (2100)	0.8	
	6M, Ave (SD)	9600 (2900)	8520 (0)	1	
ALB (g/dL)	D0, Ave (SD)	4.7 (0.4)	4.9 (0.2)	0.427	Mann–Whitney *U* test
	1M, Ave (SD)	4.5 (0.4)	4.5 (0.1)	0.498	
	3M, Ave (SD)	4.7 (0.2)	5.0 (0.1)	0.159	
	6M, Ave (SD)	4.7 (0.1)	4.6 (0)	1	

**Fig 3:**
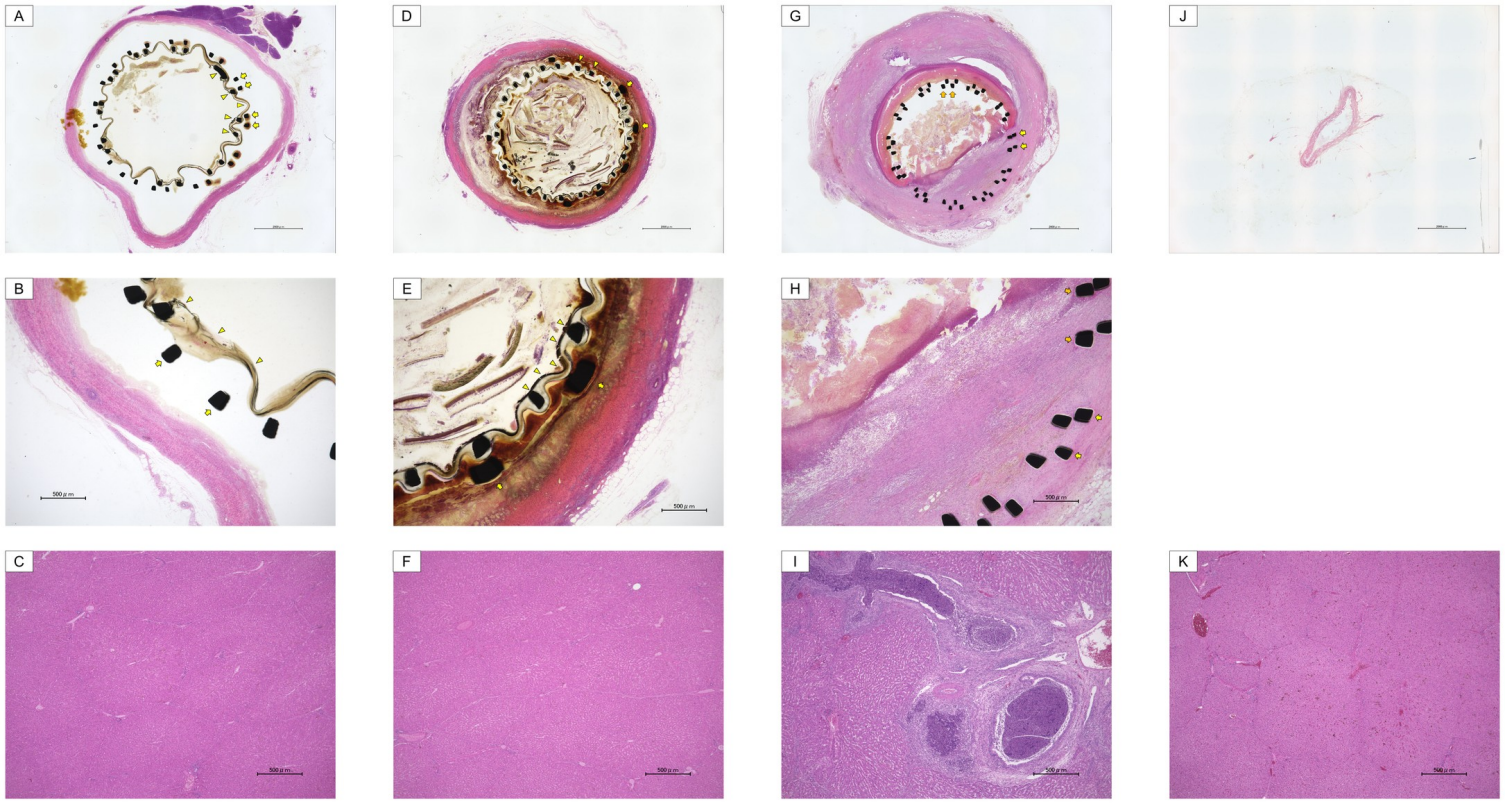
Pathological findings at 6-month follow-up in the PMEA and control groups. A–C (PMEA): Infiltration of small round cells, such as lymphocytes and plasma cells, bleeding, granulation tissue proliferation, foreign body giant cells, proliferation of mucosal epithelial cells of the bile duct and submucosal muscles, and fibrosis were not observed in the bile duct (A, B: Cole’s hematoxylin–eosin staining) and liver (C: Hematoxylin–eosin staining). The yellow arrowhead indicates the stent strut and cover membrane of the PMEA-CSEMS; and the yellow arrow indicates the stent strut of the short USEMS for migration. D–F (conventional CSEMS): Infiltration of small round cells, such as lymphocytes and plasma cells, bleeding, granulation tissue proliferation, foreign body giant cells, proliferation of mucosal epithelial cells of the bile duct and submucosal muscles, and fibrosis were not observed in the bile duct (D, E: Cole’s hematoxylin–eosin staining) and liver (F: Hematoxylin–eosin staining). The yellow arrowhead indicates the stent strut and cover membrane of the conventional CSEMS; and the yellow arrow, the stent strut of the short USEMS for migration. G–I (USEMS): The stent strut was buried in the thickened fibrous wall, and many small round cells were observed in the wall (G, H: Cole’s hematoxylin–eosin staining). An embolized bacterial mass was observed in the dilated intrahepatic bile duct. Kupffer cells and granulomas were observed in the liver parenchyma. Infiltration of inflammatory cells, fibrosis, and hyperplasia of the bile ducts were observed between the hepatic lobules at 20 days in the USEMS group (I: Hematoxylin–eosin staining). The orange arrow indicates the stent strut of the USEMS; and the yellow arrow, the stent strut of the short USEMS for migration. J–L (without ERCP): Inflammatory cell infiltration and fibrosis were not observed in the bile duct (J: Cole’s hematoxylin–eosin staining) and liver (K: Hematoxylin–eosin staining).

In the pathological evaluations of the two sections on the liver side and ampulla side of the bile duct, no inflammatory cell infiltration was observed at any of the follow-up time points in the PMEA-CSEMS and conventional CSEMS groups (Figs 2E, 3A, 3B, 3D and 3E). Mild fibrosis was observed at the 1- and 3-month time points in both groups, and no fibrosis was observed at the 6-month follow-up time point in the PMEA-CSEMS and conventional CSEMS groups (Figs 2F, 3A, 3B, 3D, 3E, S2 and S3 Figs). Compared with the PMEA-CSEMS and conventional CSEMS groups, the USEMS group showed severe inflammatory cell infiltration and mild fibrosis at 20 days (Fig 3G and 3H). Compared with the PMEA-CSEMS and conventional CSEMS groups, the control group without ERCP showed no inflammatory cell infiltration and fibrosis (Fig 3J). In the pathological evaluation of the liver, no inflammatory cell infiltration or fibrosis was observed at any of the time points in the PMEA-CSEMS and conventional CSEMS groups (Figs 2G, 2H, 3C and 3F). Compared with the PMEA-CSEMS and conventional CSEMS groups, the USEMS group showed severe inflammatory cell infiltration, mild fibrosis, and obstructive jaundice at 20 days (Fig 3I). Compared with the PMEA-CSEMS and conventional CSEMS groups, the control group without ERCP showed no inflammatory cell infiltration and fibrosis (Fig 3K).

## Discussion

Recently, several stents have been developed for preventing biliary sludge formation [13–15]. A phase-2 pilot study of an antimicrobial nanosilver-coated CSEMS reported that median stent patency was 179 days, and the primary reintervention rate was 27.3% without serious adverse events [13]; however, it should be noted that this was a small pilot study (n = 24), but with concerns about its biosafety to humans [16]. A previous study using animal models and human cell lines reported that nanosilver may cause adverse events, such as those involving the liver, lungs, and hematotoxicity [16]. PMEA was registered with the American Food and Drug Administration in 2000 (approval number, K993772). PMEA-coated circuits have been widely used in artificial heart–lung bypass, and numerous studies have demonstrated their biosafety and utility [17–20]. Furthermore, in this study, we confirmed the biosafety of PMEA-CSEMS in the swine bile duct. We believe that it may be safe for the human bile duct as well because humans and swine have similar physiological and anatomical attributes [21].

In a clinical study, Lee et al. reported that an anti-reflux valve metal stent placed to reduce duodenobiliary reflux prolonged the patency period relative to that of conventional CSEMS [14]; however, this stent has not shown efficacy in additional clinical studies [22,23]. We believe that this stent will prevent food reflux but not protein or bacterial adsorption. An in vitro study showed that PMEA suppressed protein adsorption [6], which is an early stage of sludge formation [8]. In this study, the PMEA-CSEMS did not show superiority because the conventional CSEMS did not cause liver dysfunction or jaundice. This result may be because of the short follow-up period. However, waiting until the appearance of obstructive jaundice biased the postmortem changes due to unexpected death. This type was noted to be non-inferior and safe compared with the conventional stents for at least 6 months. Therefore, we plan to evaluate its efficacy against biliary obstruction in a future clinical study. Jansen et al. reported that a hydrophilic polymer-coated (Hydromer, Inc., Somerville, N.J.) plastic stent reduced bacterial adherence relative to that observed with an uncoated stent in vitro and *in vivo* [15]; however, this stent did not show efficacy in a clinical study [24]. We believe that hydrophilic polymers are less durable because they are soluble in water. Because PMEA is a water-insoluble polymer and does not require immobilization [6], we believe that it is suitable as a surface modifier for medical devices.

Tanaka et al. reported that controlling the interfacial interactions of polymeric biomaterials with water is important for biomedical applications [25]. Those researchers hypothesized that the presence of “intermediate water,” which is hydrated water bound weakly to the polymer surface, is one of the reasons why PMEA could suppress protein adsorption. According to this hypothesis, intermediate water blocks the binding of proteins and non-freezing water, which is hydrated water bound strongly to the polymer surface [25].

In this study, we used undecalcified specimens to evaluate the histological effects of a PMEA stent on the biliary wall. Undecalcified specimens are prepared by the embedment of specimen sections in hard resin and are mainly used for bone morphometry [26,27]. Recently, undecalcified specimens have been used to evaluate the histological effects of medical materials, such as implants on tissues [28]. By utilizing resin embedding, identifying microbial colonization and its products, calcium bilirubinate, and calcium palmitate crystals may be possible. Undecalcified specimens can be evaluated without artifacts during stent removal. We showed the biosafety of PMEA-CSEMS serologically and pathologically using porcine bile ducts and undecalcified specimens. Thus, the novel polymer-coated CSEMS can be clinically applied for sludge resistance.

A limitation of this study was the small sample size owing to the application of the 3R principle (reduction, replacement, and refinement) [29]. In this study, bile viscosity measurement, component analysis, and bacterial culture were not performed because collecting bile samples without affecting the bile ducts and stents during bile duct removal was difficult. Local inflammatory findings were evaluated by pathological observation of the bile duct wall.

We demonstrated the biosafety of PMEA-CSEMS *in vivo*.

## Supporting information

S1 Fig Blood biochemistry results of the PMEA-CSEMS, conventional CSEMS, USEMS, and control without ERCP groups. (PDF)

S2 Fig Pathological findings from the long axis of the bile duct at the 6-month follow-up in the PMEA-CSEMS group. Inflammatory cell infiltration and fibrosis were not observed in the bile duct (Cole’s hematoxylin–eosin staining: Upper, liver side and lower, duodenal side).
(TIF)

S3 Fig Pathological finding from the long axis of the bile duct at the 6-month follow-up in the conventional CSEMS group. Inflammatory cell infiltration and fibrosis were not observed in the bile duct (Cole’s hematoxylin–eosin staining: Upper, liver side and lower, duodenal side).
(TIF)

S1 Table Blood biochemistry results of the PMEA-CSEMS and conventional CSEMS groups. (PDF)

S2 Table Blood biochemistry results of the USEMS and control without ERCP groups. (PDF)
