# Supplementary figures and images for: Biosafety of a novel covered self-expandable metal stent coated with poly(2-methoxyethyl acrylate) in vivo
Source: PLoS One. 2021 Sep 24;16(9):e0257828. doi: 10.1371/journal.pone.0257828 (PMC8462702; doi:10.1371/journal.pone.0257828)

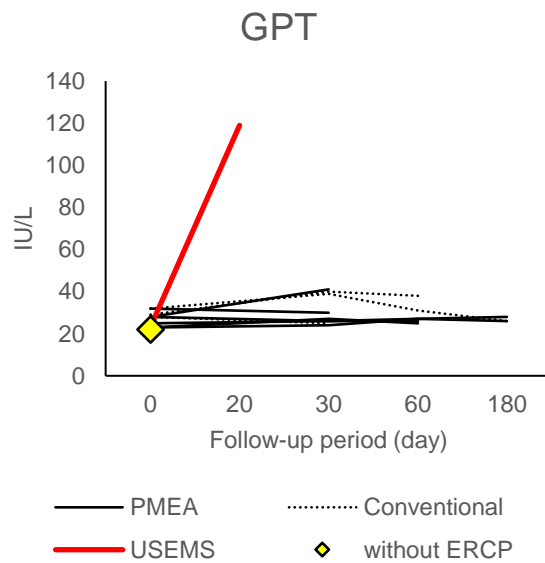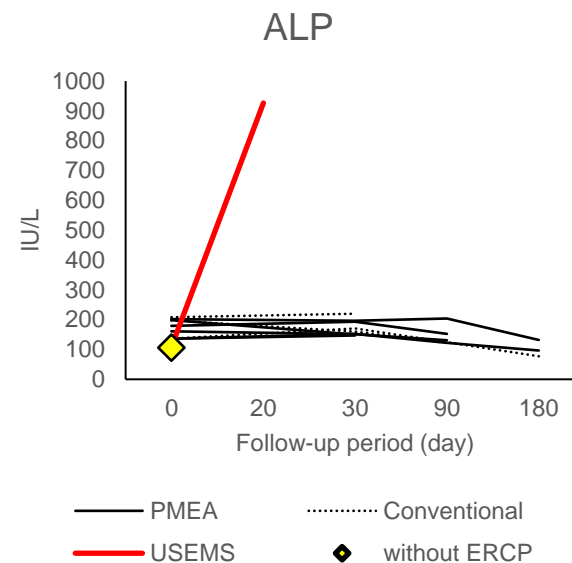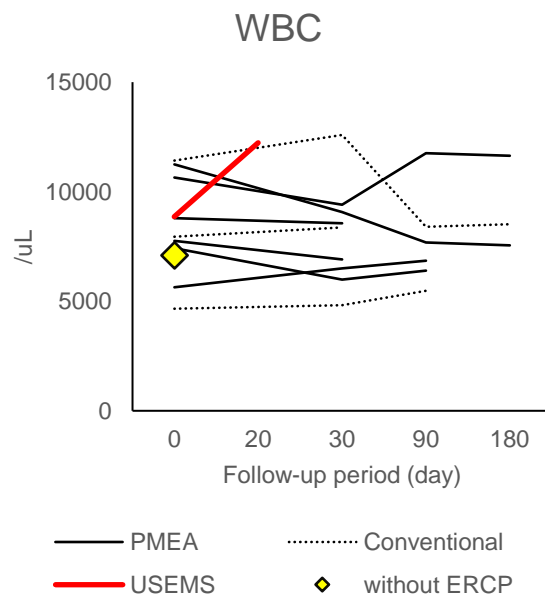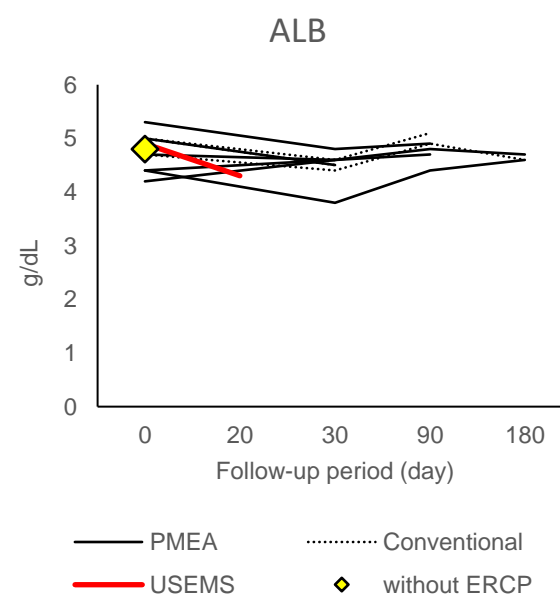

Supplement: S1 Fig — (PDF) [file pone.0257828.s001.pdf]

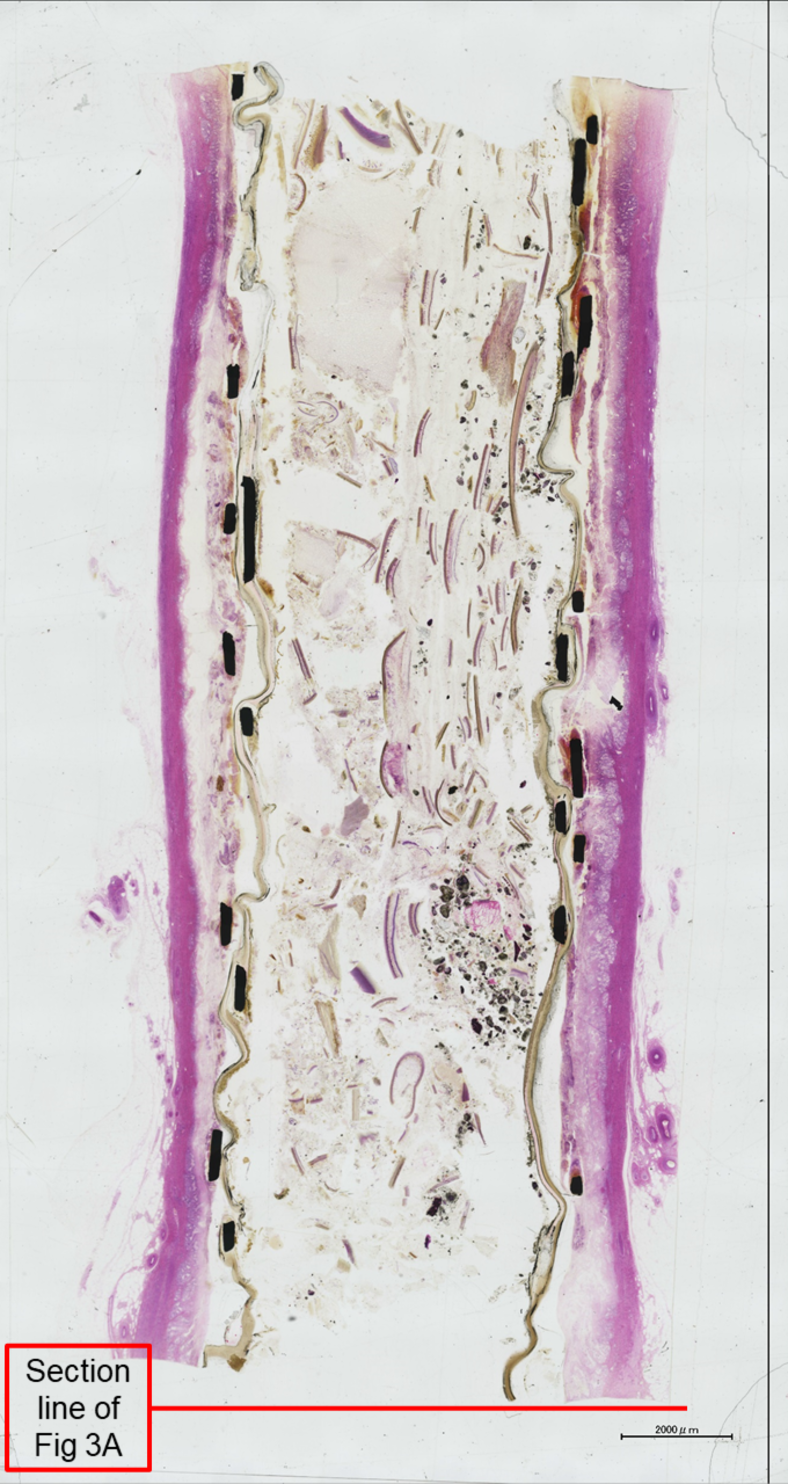

Supplement: S2 Fig — Inflammatory cell infiltration and fibrosis were not observed in the bile duct (Cole’s hematoxylin–eosin staining: Upper, liver side and lower, duodenal side). (TIF) [file pone.0257828.s002.tif]

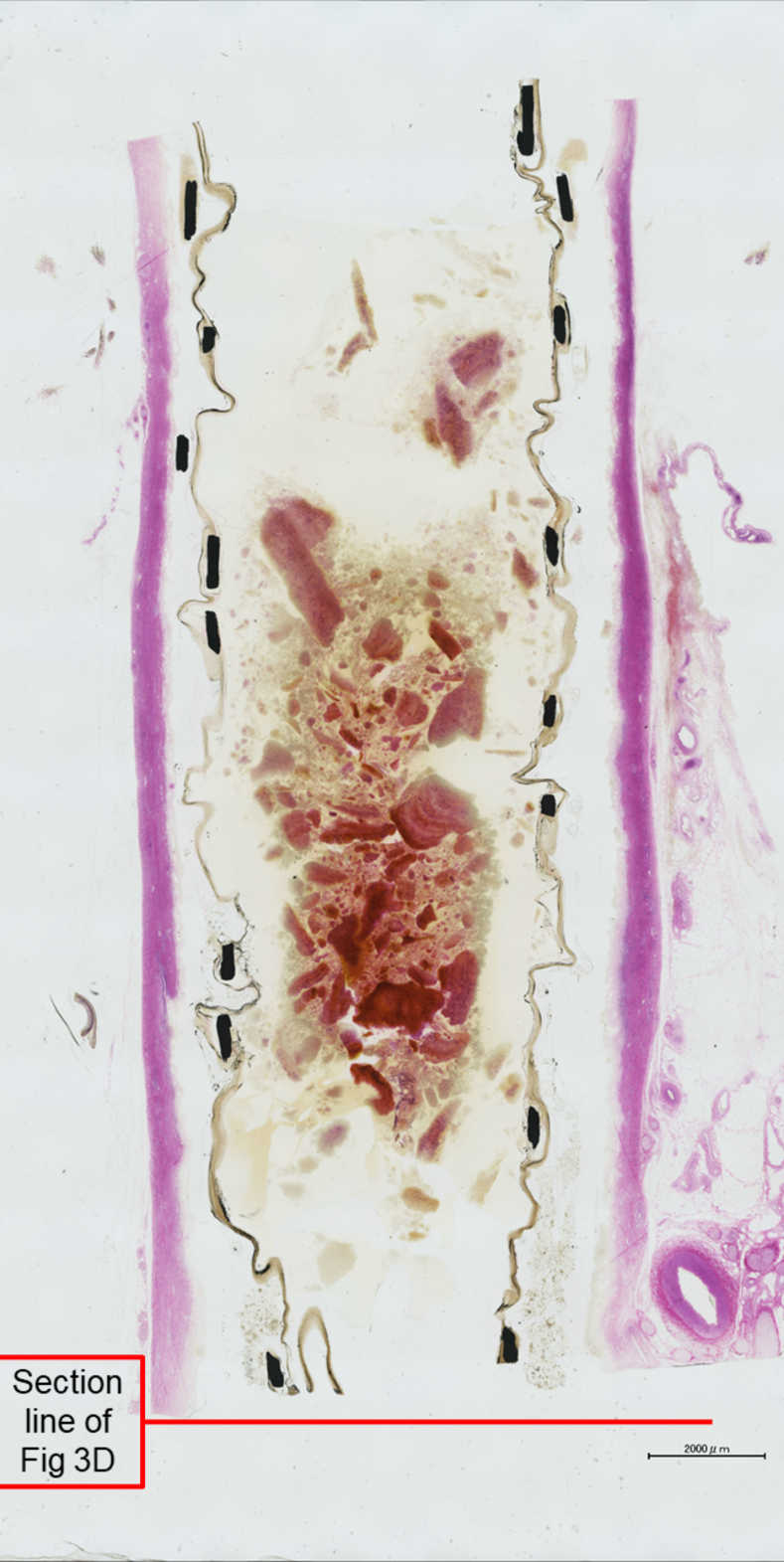

Supplement: S3 Fig — Inflammatory cell infiltration and fibrosis were not observed in the bile duct (Cole’s hematoxylin–eosin staining: Upper, liver side and lower, duodenal side). (TIF) [file pone.0257828.s003.tif]
