# Supplementary material for: Biosafety of a novel covered self-expandable metal stent coated with poly(2-methoxyethyl acrylate) in vivo
Source: PLoS One. 2021 Sep 24;16(9):e0257828. doi: 10.1371/journal.pone.0257828 (PMC8462702; doi:10.1371/journal.pone.0257828)
Supplement: S1 Table — (PDF) [file pone.0257828.s004.pdf]

glutamic pyruvic transaminase, GPT (IU/L)

No.	Stent, period	Day0	1M	3M	6M
1	PMEA 1M	28	41		
2	PMEA 1M	32	30		
3	PMEA 3M	23	27	25	
4	PMEA 3M	25	26	26	
5	PMEA 6M	28	26	27	28
6	PMEA 6M	23	24	27	26
7	Conventional 1M	28	25		
8	Conventional 3M	29	40	38	
9	Conventional 6M	32	39	31	26

alkaline phosphatase, ALP (IU/L)

No.	Stent, period	Day0	1M	3M	6M
1	PMEA 1M	199	150		
2	PMEA 1M	136	147		
3	PMEA 3M	161	151	131	
4	PMEA 3M	179	193	152	
5	PMEA 6M	136	152	122	97
6	PMEA 6M	202	197	204	132
7	Conventional 1M	208	220		
8	Conventional 3M	198	160	122	
9	Conventional 6M	136	171	125	77

white blood cell, WBC (/uL)

No.	Stent, period	Day0	1M	3M	6M
1	PMEA 1M	8790	8550		
2	PMEA 1M	7750	6900		
3	PMEA 3M	5640	6490	6840	
4	PMEA 3M	7420	5990	6400	
5	PMEA 6M	11250	9070	7680	7550
6	PMEA 6M	10650	9400	11760	11640
7	Conventional 1M	7950	8360		
8	Conventional 3M	4660	4810	5470	
9	Conventional 6M	11420	12590	8390	8520

albumin, ALB (g/dL)

No.	Stent, period	Day0	1M	3M	6M
1	PMEA 1M	5	4.5		
2	PMEA 1M	4.7	4.6		
3	PMEA 3M	5.3	4.8	4.9	
4	PMEA 3M	4.4	4.6	4.7	
5	PMEA 6M	4.4	3.8	4.4	4.6
6	PMEA 6M	4.2	4.6	4.8	4.7
7	Conventional 1M	5	4.5		
8	Conventional 3M	5	4.6	5.1	
9	Conventional 6M	4.7	4.4	4.9	4.6

Pathological evaluation of Bile duct

No.	stent, period	part	inflammation	fibrosis
1	PMEA 1M	liver side	none	none
		ampulla side	none	none
2	PMEA 1M	liver side	none	mild
		ampulla side	none	mild
3	PMEA 3M	liver side	none	mild
		ampulla side	none	mild
4	PMEA 3M	liver side	none	none
		ampulla side	none	none
5	PMEA 6M	liver side	none	none
		ampulla side	none	none
6	PMEA 6M	liver side	none	none
		ampulla side	none	none
7	Conventional 1M	liver side	none	none
		ampulla side	none	mild
8	Conventional 3M	liver side	none	none
		ampulla side	none	none
9	Conventional 6M	liver side	none	none
		ampulla side	none	none

Pathological evaluation of Liver

No.	stent, period	inflammation	fibrosis
1	PMEA 1M	none	none
2	PMEA 1M	none	none
3	PMEA 3M	none	none
4	PMEA 3M	none	none
5	PMEA 6M	none	none
6	PMEA 6M	none	none
7	Conventional 1M	none	none
8	Conventional 3M	none	none
9	Conventional 6M	none	none
