# Supplementary material for: Biosafety of a novel covered self-expandable metal stent coated with poly(2-methoxyethyl acrylate) in vivo
Source: PLoS One. 2021 Sep 24;16(9):e0257828. doi: 10.1371/journal.pone.0257828 (PMC8462702; doi:10.1371/journal.pone.0257828)
Supplement: S2 Table — (PDF) [file pone.0257828.s005.pdf]

cases	unit	D0	D20
USEMS (in USEMS)	GPT (IU/L)	23	119
	ALP (IU/L)	110	927
	WBC (/dL)	8850	12230
	ALB (g/dL)	4.9	4.3
without ERCP	GPT (IU/L)	22	
	ALP (IU/L)	106	
	WBC (/dL)	7090	
	ALB (g/dL)	4.8	
